# Understanding the Facet Joint Fusion Process: A Narrative Review for Clinicians and Surgeons Addressing the Who, Why, What, When, Where, Which, and How

**DOI:** 10.7759/cureus.111611

**Published:** 2026-06-27

**Authors:** Carla García-Ramos, Diana Laura Hernandez Moctezuma, Maria-Fernanda Medina-Perez, Aisha-Alexandra Murillo-Chavez, Diego-Alberto Nuñez-Arreola, Alejandro Antonio Reyes-Sanchez, Irving O Estevez-Garcia, Ernesto Roldan-Valadez

**Affiliations:** 1 Spine Surgery, Instituto Nacional de Rehabilitación “Luis Guillermo Ibarra Ibarra”, Mexico City, MEX; 2 Spine Surgery Research, Instituto Nacional de Rehabilitación, Mexico City, MEX; 3 Spine Surgery, Instituto Nacional de Rehabilitación "Luis Guillermo Ibarra Ibarra", Mexico City, MEX; 4 Research, Instituto Nacional de Rehabilitación "Luis Guillermo Ibarra Ibarra", Mexico City, MEX; 5 Spine Surgery, National Institute of Rehabilitation Luis Guillermo Ibarra Ibarra, Mexico City, MEX; 6 Research, Instituto Nacional de Rehabilitacion "Luis Guillermo Ibarra Ibarra", Mexico City, MEX; 7 Radiology, I.M. Sechenov First Moscow State Medical University, Moscow, RUS

**Keywords:** degenerative spondylolisthesis, facet joint fusion, fusion rates, lumbar spine, postoperative care, spinal stability, surgical management

## Abstract

Lumbar facet joint fusion has established itself as an important aspect of the surgical management of degenerative spondylolisthesis (DS) and related pathological conditions of the spine. Understanding the fusion process and its clinical implications is essential for optimizing patient outcomes, particularly in the context of advancing surgical techniques and imaging modalities.

This narrative review aims to assess the existing evidence regarding facet joint fusion in the lumbar spine through the structured framework of WHO, WHY, WHAT, WHEN, WHERE, WHICH, and HOW.

A structured search of PubMed/MEDLINE-a freely and globally accessible database, selected so that the search can be reproduced by any reader without licensed or subscription-based access-was conducted from inception to the date of the search (15 June 2026), combining the MeSH terms "Spinal Fusion", "Spondylolisthesis", "Lumbar Vertebrae", and "Zygapophyseal Joint" with free-text variants of facet joint fusion and the principal interbody techniques. Peer-reviewed studies, consensus guidelines, and clinical trials addressing facet joint fusion, fusion rates, and postoperative outcomes were included.

Facet joint fusion contributes significantly to spinal stability and favorable long-term results in DS. Patient selection (WHO), pathophysiological understanding (WHY), and surgical techniques (WHAT) - anterior lumbar interbody fusion (ALIF), oblique lateral interbody fusion (OLIF), extreme lateral interbody fusion (XLIF), posterior lumbar interbody fusion (PLIF), and transforaminal lumbar interbody fusion (TLIF) - are pivotal. Timing (WHEN), anatomical considerations (WHERE), and choice of operative and imaging methods (WHICH) influence success rates. Optimal postoperative care (HOW) is associated with reduced reoperation rates and improved quality of life.

Lumbar spinal DS surgery with facet joint fusion is a complex, multifactorial process requiring careful planning, execution, and management in the dynamic process of perioperative care. This review provides a modern perspective for clinicians in the evidence-based care of this patient population.

## Introduction and background

Lumbar degenerative spondylolisthesis (DS) is a prevalent spinal pathology characterized by anterior displacement of one vertebral body over the one below, most commonly at the L4-L5 segment. It arises from degeneration of the intervertebral discs, facet joints, and supporting ligaments, resulting in spinal stenosis, instability, and neurologic compromise [1,2]. Affected patients typically report low back pain, neurogenic claudication, and radiculopathy that impair mobility and quality of life [1,3].

Spinal decompression is the principal procedure performed in DS to relieve neural compression. Yet, debate persists regarding the added value of fusion and instrumentation, which aim to stabilize the affected segments and prevent further displacement or instability [4-6]. The main interbody techniques - anterior lumbar interbody fusion (ALIF), oblique lateral interbody fusion (OLIF), extreme lateral interbody fusion (XLIF), posterior lumbar interbody fusion (PLIF), and transforaminal lumbar interbody fusion (TLIF) - all seek solid bony union of the vertebral bodies to relieve symptoms and restore stability [7].

Fusion status has increasingly been recognized as a determinant of surgical success and patient-reported outcomes, and radiological assessment of fusion is central to judging the long-term efficacy of the intervention, as solid fusion is associated with greater patient satisfaction and fewer reoperations [8]. Facet joint fusion, in particular, has emerged as a factor associated with long-term clinical outcomes and spinal stability; however, current evidence primarily reflects an association rather than a proven causal relationship, underscoring the need for a comprehensive understanding of the fusion process across all involved anatomical sites [9,10].

This review synthesizes the current evidence on fusion in lumbar spine surgery for DS, organized around seven structured questions: WHO (patient characteristics), WHY (pathophysiological and biomechanical rationale), WHAT (surgical techniques and fusion strategies), WHEN (timing and indications for surgery), WHERE (anatomical sites and radiological assessment), WHICH (optimal surgical and radiological methods), and HOW (practical clinical implementation and postoperative care). By clarifying these aspects, the review aims to strengthen clinicians’ understanding, optimize surgical decision-making, and improve patient care.

## Review

Methods

This narrative review utilized the SANRA (Scale for the Assessment of Narrative Review Articles) reporting framework. To ensure that the evidence base remains freely and globally accessible and that any reader can reproduce the search without licensed or subscription-based access, we performed the literature search in PubMed/MEDLINE, a publicly accessible database, rather than in subscription-restricted platforms. The literature search had no year restrictions, and language restrictions were limited to English and Spanish. The search combined controlled vocabulary (MeSH/Emtree) and free-text terms covering “lumbar degenerative spondylolisthesis”, “facet joint fusion”, “spinal fusion”, “ALIF”, “OLIF”, “XLIF”, “TLIF”, “PLIF”, “posterolateral fusion”, “fusion rate”, “pseudarthrosis”, and “imaging assessment”.

The inclusion criteria included peer-reviewed primary studies (randomized controlled trials, prospective and retrospective cohort studies, case series (with ≥20 patients)), systematic reviews, meta-analyses, consensus statements, and authoritative narrative reviews addressing biomechanical, surgical, radiological, or clinical outcomes of lumbar fusion for DS and related conditions. Reference lists of pivotal articles were hand-searched to identify additional sources. Studies were excluded if they focused only on cervical or strictly thoracic pathology, did not provide any fusion-related outcomes, or were published only as conference abstracts with no full publication.

Two reviewers independently screened titles and abstracts; discrepancies were resolved through discussion until consensus was reached. To give an indicative account of the evidence base, a focused PubMed/MEDLINE query combining facet joint fusion, lumbar interbody and posterolateral fusion, and degenerative spondylolisthesis or lumbar spinal stenosis, run on the date of the search (15 June 2026) for the period 2000 to 2026, returned approximately 2,200 records; narrowing to studies addressing facet joint fusion in the lumbar spine yielded roughly 400 titles and abstracts, of which about 65 dealt specifically with facet joint fusion. After screening for relevance and prioritizing the most authoritative and recent sources, 47 studies were retained for the narrative synthesis. Because this is a narrative review (SANRA) rather than a systematic review, these figures are indicative rather than an exhaustive, fully reproducible enumeration. By the nature of this article type, the literature evolves continually, so the precise counts are expected to change over time. A formal risk-of-bias assessment is not mandated for narrative reviews under the SANRA framework; nonetheless, study quality was appraised qualitatively, giving greater interpretive weight to randomized controlled trials, prospective cohorts, and systematic reviews than to retrospective series or small case reports. The final corpus prioritized publications from 2020-2026 to capture the most recent literature and current practice, while seminal earlier works were retained when they were the most authoritative source on a specific concept (e.g., the Kirkaldy-Willis degenerative cascade). Data were then organized into a table by study type, surgical technique, fusion rate, complications, and patient-reported outcome measures, and synthesized narratively according to the seven domains (WHO, WHY, WHAT, WHEN, WHERE, WHICH, HOW) that structure and guide this review.

WHO: patient characteristics and clinical presentation

Symptoms of degenerative spondylolisthesis begin to emerge more frequently in those aged in their sixties and seventies and are more common in females, especially in postmenopausal women. The increasing occurrence in females is hypothesized to be due to hormonal changes that lead to ligamentous laxity and accelerated degenerative processes affecting spinal stability. Increased age remains a major risk factor because of cumulative degenerative changes affecting the intervertebral discs and facet joints, compounded by reduced muscle strength and bone mineral density [2,11,12].

The clinical presentation of patients with DS typically includes many forms of low back pain and neurogenic claudication. Neurogenic claudication includes pain, weakness, or sensory changes in the lower extremities that occur when the patient is standing for long periods or walking. Radicular symptoms may result from direct nerve root compression, manifesting as sciatica, paresthesias, or motor weakness [3]. Significant symptoms can lead to a loss of self-efficacy across all aspects of life, including work and activities of daily living [8].

Reduced lumbar lordosis, paravertebral muscle spasms, and tenderness in the affected segments of the spine, as well as reduced lumbar flexion or extension, are common findings of the clinical examination. Neurologic examination may demonstrate diminished reflexes, dermatomal sensory deficits, or motor weakness, particularly affecting the L4 and L5 nerve roots. The severity of symptoms and signs, and their characteristic presentations, are typically proportional to the degree of spondylolisthesis and spinal stenosis of the lumbar spine, as seen on imaging studies [1,5].

The presence of multiple, concurrent medical conditions such as osteoporosis, obesity, diabetes mellitus, and cardiovascular disease is common in this population, adding complexity to clinical management, surgical planning, and postoperative rehabilitation [12,13]. Recent data from geriatric meta-analyses suggest that minimally invasive transforaminal lumbar interbody fusion (MIS-TLIF) surgeries yield positive results regarding survivability and quality of life in this age group (aged 65 and above) [14]. These results indicate that the identified patient cohort should be included. It is important to identify and manage coexisting conditions, as they significantly influence the surgical outcomes, fusion rates, and the risk of complications (Table 1).

**Table 1:** Demographic and clinical factors influencing lumbar fusion success. This table summarizes key patient-related factors that affect lumbar fusion outcomes, including age, sex, comorbidities, lifestyle habits, and nutritional status. Understanding these variables allows clinicians to optimize preoperative planning, risk stratification, and postoperative management to enhance fusion success rates. Source: Adapted by the authors from references [2,12,13,15]. Abbreviations: BMI, body mass index; DEXA, dual-energy X-ray absorptiometry.

Factor	Impact on fusion success	Notes
Age	Advanced age is associated with delayed bone healing and lower fusion rates.	Elderly patients may benefit from bone-stimulating adjuncts.
Sex	Postmenopausal women have an increased risk owing to osteoporosis.	Hormonal status influences bone turnover and density.
Diabetes mellitus	Impaired bone metabolism and increased risk of pseudarthrosis.	Tight glycemic control is recommended perioperatively.
Osteoporosis	Poor bone quality reduces implant fixation and fusion potential.	Preoperative DEXA screening advised; bisphosphonates or anabolic agents may help.
Smoking status	Strong negative predictor of fusion; nicotine impairs osteoblast activity and vascularity.	Smoking cessation at least 6–8 weeks preoperatively improves outcomes.
Obesity (BMI > 30)	Increased surgical complexity and risk of mechanical failure.	Tailored surgical planning and reinforced instrumentation may be necessary.
Physical activity level	Excessive early postoperative loading can compromise fusion; appropriate rehabilitation promotes long-term success.	Gradual activity increase with physiotherapy is key to avoiding stress on the fusion site.
Vitamin D deficiency	Reduces bone mineralization and healing potential.	Supplementation should be considered preoperatively in deficient patients.

**Figure 1:**
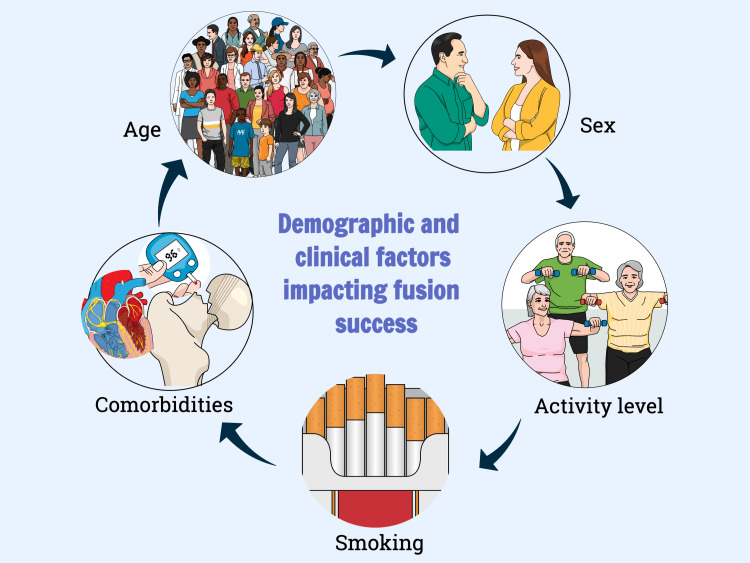
Demographic and clinical factors influencing lumbar fusion success. Key patient-related factors associated with the success of lumbar fusion procedures. Age, sex, comorbidities (such as diabetes mellitus and osteoporosis), smoking status, and baseline physical activity level play significant roles in determining fusion outcomes and complication risk. Tailored preoperative optimization strategies targeting these variables may enhance fusion rates and postoperative recovery. Image created using the Mind the Graph platform (www.mindthegraph.com), account paid by the corresponding author (E.R.V.). The original schematic has not been reproduced from previous publications, and no third-party permissions were required. No artificial intelligence tools were used in the generation or the writing of this article; the diagrams were completely conceptualized and designed by the authors.

A patient’s demographics and the comorbidities they present with should be analyzed closely to develop a personalized treatment approach, select the appropriate patient for the intervention, and achieve the best results (Figure 1).

WHY: pathophysiological and biomechanical rationale

Degenerative spondylolisthesis is caused by degeneration of spinal structural components essential for maintaining spinal stability, primarily the intervertebral discs, facet joints, and spinal ligaments. The pathophysiology is multifactorial and involves age-related degeneration of discs, specifically, dehydration, loss of height, and subsequent alterations of the lumbar spine biomechanical load. Such modifications to the lumbar spine’s biomechanical load result in facet joint stress, leading to hypertrophy, capsular laxity, and arthritic remodeling, which eventually compromise segmental stability and promote vertebral displacement [1,2].

Facet joints serve as the primary stabilizing structures of the lumbar spine and are critical to the pathogenesis of DS. They limit axial rotation, flexion-extension, and shearing forces between vertebral segments. Once degenerative changes occur in the facet joints, they become mechanically inefficient and incapable of resisting physiological loads, resulting in microinstability. Microinstability leads to compensatory thickening of joint structures, specifically ligaments, and hypertrophy of the joint capsule, and the formation of bony outgrowths that contribute to narrowing of the neural foramina and the spinal canal [9].

From a biomechanical perspective, DS primarily involves the L4-L5 spinal segment due to the unique transitional biomechanical features of this region, where lumbar lordosis is diminished, and the posterior lumbar spinal segments of L4-L5 are subjected to higher axial loads. The posterior arch and facet joints of L4-L5 experience the greatest shearing and rotational stresses during daily living activities, thereby predisposing this region to advanced degeneration [3,5].

Alterations in sagittal alignment of the spine exacerbate biomechanical vulnerabilities. Diminishing the height of lumbar intervertebral discs and lumbar lordosis changes the distribution of load to the posterior spinal structures. Consequently, this induces facet joint stress and arthropathy. Simultaneously, the stress and load applied to the lumbar spine are shifted to the anterior structures, increasing segmental translational movement and slippage, thereby increasing the load on neural structures and causing neural compression and worsening symptoms [7].

The pathophysiology and biomechanics of specific areas are needed to fully understand the rationale for spinal fusion therapies aimed at restoring lumbar segmental stability. The aim of surgical fusion is the loss of abnormal motion, redistribution of mechanical forces more evenly, and the possible reduction of further deterioration of the spinal structures. Knowledge of the underlying mechanisms improves surgical planning and clinical decision-making, thereby increasing surgical success and patient outcomes [8,9].

WHAT: surgical techniques and fusion strategies

There are many surgical procedures used for spinal fusion, for which there are different methods, instruments, and biological techniques to stabilize the lumbar segment affected by DS. They are all highly specialized, and the technique used will impact the level of fusion achieved. Understanding the nuances of each is crucial when tailoring interventions to individual patients and optimizing fusion rates.

*Anterior lumbar interbody fusion (ALIF)* uses an anterior approach to the lumbar spine and is often preferred to restore lumbar lordosis and disc height. The anterior approach allows the surgeon to see the entire lumbar spine and use larger interbody cages. Larger cages and grafts improve biomechanical stability and fusion rates. A tradeoff of improving the fusion area is the increased likelihood of injury to large anterior lumbar vascular structures, as well as the possible loss of the ability to achieve ejaculation in male patients, which limits its suitability for specific subsets and anatomical configurations [4,7]. The most recent systematic reviews of the outcomes of laparoscopic and robotic-assisted ALIF cite comparable outcomes, yet perioperative morbidity is reduced. Longitudinal studies and the economic impact of this approach have yet to be established [16].

*Oblique lateral interbody fusion (OLIF) *accesses the lumbar disc space without traversing the spinal canal. By avoiding direct posterior neural manipulation and paraspinal muscle stripping, OLIF may reduce approach-related morbidity while still allowing wide disc-space preparation and placement of a relatively large interbody cage. OLIF provides lateral disc space preparation and insertion of large-capacity interbody devices, restoring disc height and foraminal height and providing segmental stability. OLIF also indirectly decompresses neural tissue and supports the correction of segmental deformation in the sagittal and coronal planes. OLIF also significantly improves leg pain and provides meaningful reductions in the visual analog scale score and the Oswestry Disability Index score, which compare favorably with other techniques [17,18].

*Extreme lateral interbody fusion (XLIF),* on the other hand, approaches the lumbar disc via a lateral retroperitoneal transpsoas corridor. This process sidesteps posterior canal entry and supports indirect foraminal decompression and correction of coronal plane deformity. The procedure can be used generally from T12/L1 to L4/L5. Still, it cannot be used on L5/S1 due to iliac crest obstruction and the more anterior courses of the lumbar plexus and iliac vessels in the caudal lumbar region. Intraoperative neuromonitoring is considered essential because the approach traverses the psoas muscle near the lumbar plexus. This approach requires access to the psoas muscle, which is near the lumbar plexus; therefore, intraoperative neuromonitoring is essential and is also reserved for patients in whom indirect decompression is sufficient and preoperative imaging confirms a safe lateral corridor [19,20].

*Transforaminal lumbar interbody fusion (TLIF)* allows interbody access through a unilateral facetectomy and minimizes neural retraction compared with PLIF. TLIF exhibits similar fusion rates and comparable symptom relief while incurring fewer complications related to neural manipulation. It also preserves contralateral facet integrity, contributing to postoperative spinal stability and a lower incidence of adjacent segment disease [7]. TLIF variants using emerging biportal endoscopic techniques add to these benefits by further reducing soft-tissue trauma and shortening postoperative recovery, with reported outcomes comparable to those of conventional MIS-TLIF [21].

*Posterior lumbar interbody fusion (PLIF) *remains widely used. It begins with posterior decompression of the area of interest, followed by insertion of interbody cages filled with autograft or synthetic osteoinductive material. PLIF offers direct neural decompression with anterior column support, which increases column and disc stability and supports disc height restoration. This approach, however, manipulates neural tissue, causing greater morbidity and complications, including dural tears with nerve-root injuries [2,7].

*Posterolateral fusion (PLF)* is a common adjunctive strategy in which a bone graft is placed on the facet joint and the transverse process. This procedure can be performed on its own or as a supplement to decompression. PLF has lower fusion rates than other interbody techniques; however, due to its short operative time and lower complication rate, PLF is justified, especially in the elderly or fragile population [11,22].

Emerging research indicates that combining traditional techniques with targeted facet joint fusion can be beneficial. A review of the long-term outcomes of instrumented facet fusion noted an approximate 92% success rate with a fusion and a low rate of revision. This rate supports the facet-focused strategies as a fusion approach that is both durable and minimally invasive [10]. Achieving direct facet joint fusion may significantly enhance segmental rigidity and overall stability, improving fusion rates and clinical outcomes. Novel biologic agents, such as bone morphogenetic proteins (BMPs), have been explored with promising results for facet joint and posterolateral fusion, particularly in elderly or osteoporotic patients with impaired bone healing potential [9,11,23,24].

**Figure 2:**
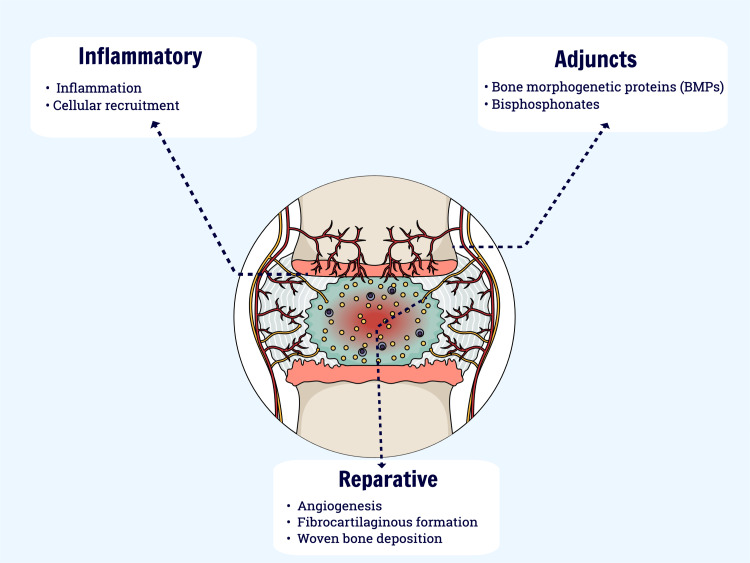
Bone-healing cascade underpinning lumbar fusion success. Schematic of the phased bone-healing process in lumbar fusion surgery. The inflammatory phase (hematoma formation and cellular recruitment) transitions to the reparative phase (angiogenesis, fibrocartilaginous callus formation, and woven-bone deposition), culminating in the remodeling phase with mature lamellar-bone formation. Pharmacologic and biologic adjuncts may modulate these stages to improve fusion rates. Image created using the Mind the Graph platform (www.mindthegraph.com), account paid by the corresponding author E.R.V. The original schematic has not been reproduced from previous publications, and no third-party permissions were required. No artificial intelligence tools were used in the generation or the writing of this article; the diagrams were completely conceptualized and designed by the authors. Abbreviations: BMPs, bone morphogenetic proteins.

**Figure 3:**
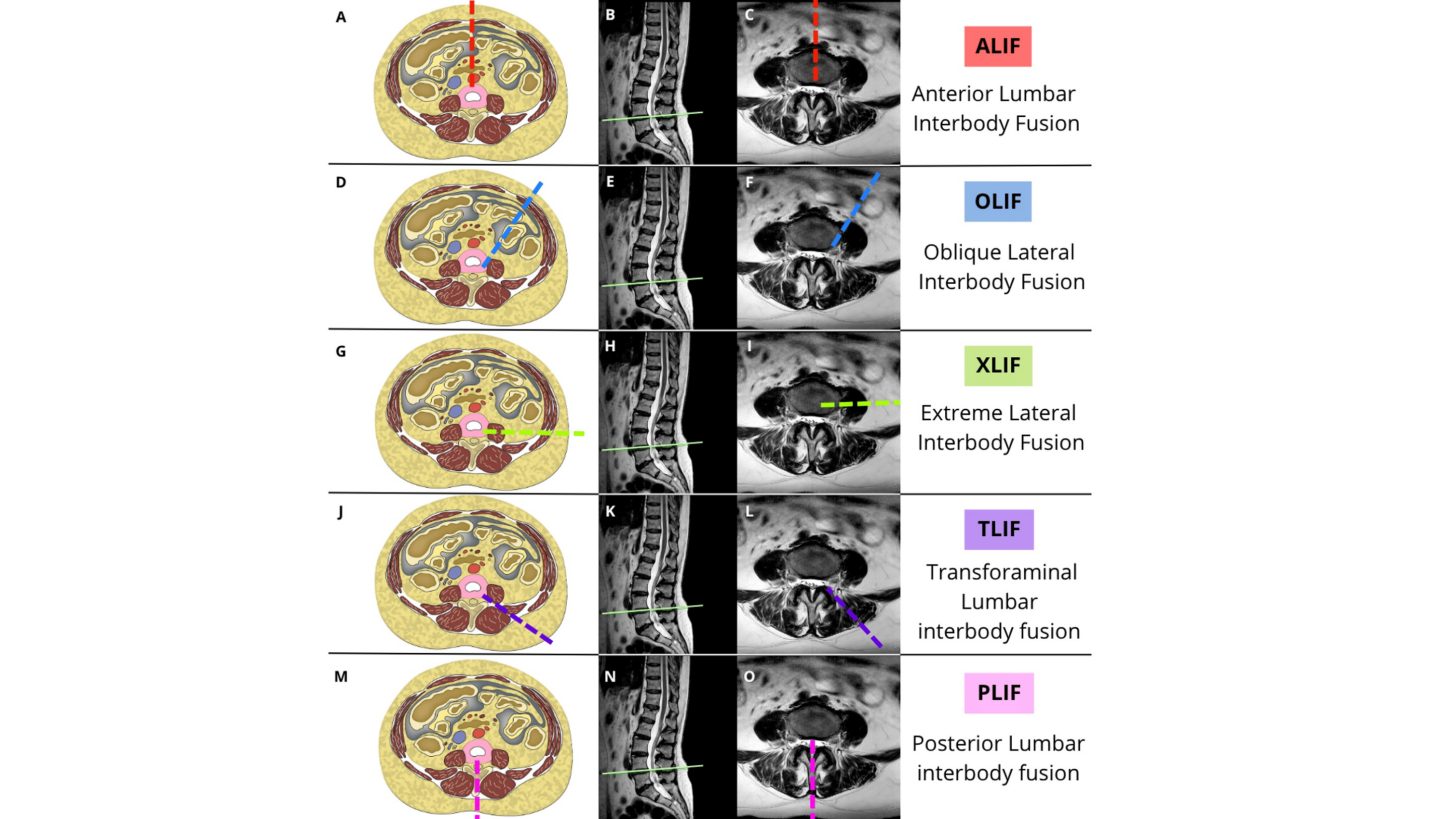
Approaches in lumbar spine surgery and magnetic resonance imaging correlation. For each technique, the left panel shows the axial diagram, the middle panel the lumbar sagittal MRI showing the target level, and the right panel the lumbar axial MRI showing the access window. Panels A-C, anterior lumbar interbody fusion; panels D-F, oblique lumbar interbody fusion; panels G-I, extreme lateral/transpsoas lumbar interbody fusion; panels J-L, transforaminal lumbar interbody fusion; panels M-O, posterior lumbar interbody fusion. Image created using the Mind the Graph platform (www.mindthegraph.com), account paid by the corresponding author E.R.V. The original schematic has not been reproduced from previous publications, and no third-party permissions were required. No artificial intelligence tools were used in the generation or the writing of this article; the diagrams were completely conceptualized and designed by the authors. Abbreviations: ALIF, anterior lumbar interbody fusion; MRI, magnetic resonance imaging; OLIF, oblique lateral interbody fusion; PLIF, posterior lumbar interbody fusion; TLIF, transforaminal lumbar interbody fusion; XLIF, extreme lateral interbody fusion.

Figure 2 demonstrates the bone-healing cascade, and Figure 3 demonstrates surgical approaches with MRI correlations.

Each fusion strategy has unique advantages and limitations. Patient-specific factors, including age, bone quality, degree of spinal instability, comorbidities, and anatomical complexity, must inform the surgeon’s choice of technique to achieve optimal fusion outcomes and improved quality of life (Table 2) [5,8].

**Table 2:** Comparison of lumbar fusion techniques (PLIF, TLIF, ALIF, OLIF, XLIF, and PLF). Comparison of the principal lumbar fusion techniques highlighting surgical indications, advantages, disadvantages, reported fusion rates, and complication profiles. The table provides clinicians and surgeons with a concise reference for selecting the most appropriate technique based on patient and disease characteristics. Reported fusion rates correspond to the ranges described in the cited systematic reviews and meta-analyses for one- and two-level surgery at 6-24-month follow-up. Source: Adapted by the authors from references [7,10,16-21,25-31]. Abbreviations: ALIF, anterior lumbar interbody fusion; ATP, anterior-to-psoas; CSF, cerebrospinal fluid; ODI, Oswestry Disability Index; OLIF, oblique lateral interbody fusion; PLF, posterolateral fusion; PLIF, posterior lumbar interbody fusion; TLIF, transforaminal lumbar interbody fusion; XLIF, extreme lateral interbody fusion.

Technique	Surgical indications	Advantages	Disadvantages	Reported fusion rate	Common complications
PLIF	Disc degeneration, spondylolisthesis, instability	Bilateral access; good disc-space restoration	Higher risk of dural tear; neural element manipulation	~85-95%	Neural injury, CSF leak, epidural fibrosis
TLIF	Similar to PLIF; preferred in unilateral pathology	Less neural retraction; unilateral approach; good disc height	Limited contralateral visualization; potential cage migration	~88-95%	Cage subsidence, radiculopathy
ALIF	Isolated disc disease, sagittal-balance restoration	Avoids posterior muscles; large cage placement possible	Risk to vascular structures; retrograde ejaculation (males)	~90-96%	Vascular injury, ileus, retroperitoneal hematoma
PLF	Less severe instability; adjunct to decompression	Simpler; avoids disc space; shorter operative time	Lower fusion rates; less sagittal-balance correction	~70-85%	Pseudarthrosis, hardware failure
OLIF/ATP	Restoration of disc height, indirect decompression, sagittal correction	Preservation of psoas and lumbar plexus; minimally invasive; better early back pain/ODI than TLIF/PLIF	Abdominal/vascular injury, sympathetic chain irritation, hip-flexor weakness	~90-96%	Cage subsidence, sympathetic lesion, pseudarthrosis
XLIF	Degenerative disc disease, indirect decompression in foraminal stenosis, low-grade spondylolisthesis	Better sagittal correction, indirect decompression	Transpsoas dissection may cause neurologic deficits	~85-93%	Transient neurologic deficits, lumbar plexus lesion
Facet joint fusion (instrumented)	Adjunct to interbody/posterolateral fusion; degenerative segments	Direct stabilisation; minimally invasive; durable long-term results	Lower standalone fusion potential without interbody support	~92%	Screw loosening, adjacent segment disease

It should be emphasized that reported fusion rates vary substantially across studies, depending on the imaging criteria used to define fusion, the duration of follow-up, patient selection, and the use of biologic adjuncts; the comparative values summarized here should therefore be interpreted in light of this heterogeneity.

WHEN: indications, timing, and decision-making for surgical intervention

Surgical intervention seeks optimal timing and indications to achieve effective clinical outcomes and enhance quality of life. When determining the timing of surgical intervention, balance the patient’s presenting symptoms with imaging findings, the level of functional impairment, and the inadequacy of conservative therapies.

The most prominent indications for lumbar spinal fusion surgery in DS include persistent neurogenic claudication, intractable radicular pain, progressive neurologic deficits, and significant functional impairment. Surgery is generally warranted when conservative measures (i.e., physical therapy, analgesics, and epidural steroid injections) provide insufficient relief of the aforementioned symptoms for a period of at least three to six months [2,3]. The latest 2024-2025 meta-analyses suggest that for certain patients with low-grade DS, decompression alone, compared to decompression supplemented by fusion, is unlikely to benefit patients more than the latter, thus indicating individualized management rather than a blanket policy on the necessity for fusion [6].

There are circumstances in which surgical intervention before the three- to six-month period may be justified. The acute onset and progressive worsening of a neurologic deficit, including motor weakness, foot drop, bowel and bladder dysfunction, and pain that is threshold-refractory to all modalities, may be considered an absolute indication to perform decompression and stabilization. Surgical intervention during the early postoperative phase is thought to prevent permanent nerve injury and restore the patient’s long-term functional capability [4,5].

For many patients, age, comorbidities, and the level of function all influence the optimal treatment course. Younger patients with significant pain and functional impairment are good candidates for intervention to promote stability and reduce progressive impairment. The risks and benefits of intervention should be assessed for older patients and those with multiple comorbidities. Modern studies have documented improved function in elderly patients with fusion when enhanced perioperative protocols are used [11,14,22]. A 2025 network meta-analysis directly comparing decompression alone, decompression plus dynamic stabilization, and decompression plus fusion found broadly similar improvements in pain and disability across techniques, while highlighting differences in operative time and blood loss that should inform technique selection [32].

Surgical decision-making in DS is multifactorial, integrating the patient’s symptom burden, neurological status, comorbidities, and response to conservative care rather than relying on any single radiographic threshold. Imaging provides important supporting evidence - for example, vertebral slippage (often around 4-5 mm), flexible or dynamic instability on flexion-extension views, and significant narrowing of the foramen or central canal producing foraminal and/or central stenosis. Advanced imaging (MRI and CT) is key to objectively characterizing these findings and, together with the overall clinical picture, informs both surgical urgency and procedural planning [1,9].

Emerging evidence also supports earlier intervention in patients demonstrating significant segmental instability or rapidly progressive symptoms to avoid further degenerative change, adjacent segment disease, and persistent chronic pain. Early stabilization may halt disease progression and improve outcomes by promoting more favorable fusion conditions [7,8]. Surgical decision-making in DS should ultimately be individualized and guided by a comprehensive assessment of clinical presentation, patient preferences, imaging findings, and anticipated functional outcomes.

WHERE: anatomical considerations and radiological assessment

A detailed anatomical understanding of releasable structures and a highly skilled approach to assessing fusion status in the postoperative period are essential for the successful application of lumbar fusion in deformity surgery. The anatomy of the structures in the intervertebral space does not stop at the limit of the disc space. Still, it includes the facet joints, ligament complexes, and the neural elements that surround and embrace the structures, providing stabilization of the spinal column.

**Figure 4:**
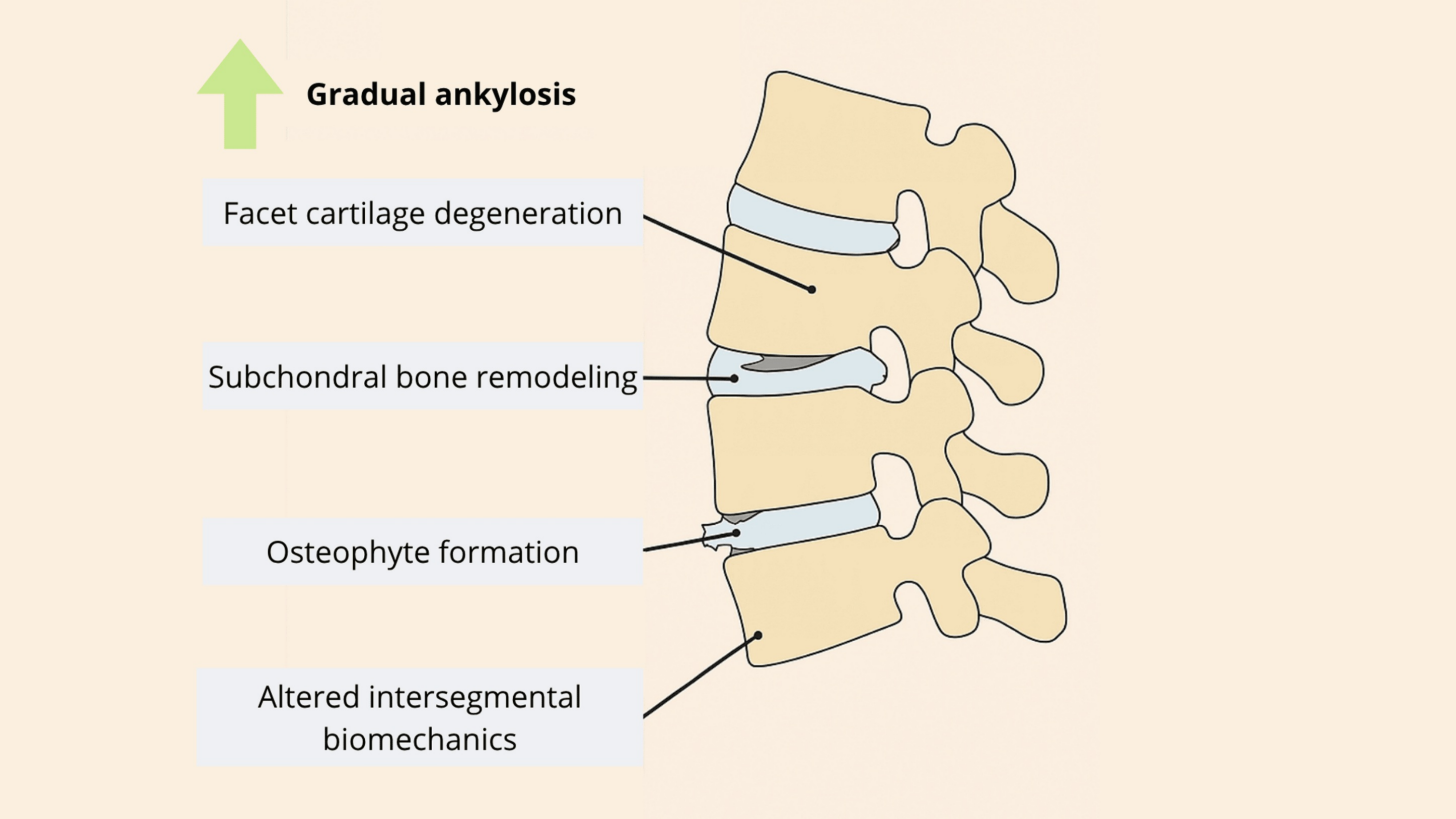
Pathophysiological mechanisms of facet joint fusion in lumbar degenerative spondylolisthesis. Proposed pathophysiological cascade leading to spontaneous facet joint fusion: progressive degeneration of the facet joint cartilage, subchondral bone remodeling, osteophyte formation, and eventual ankylosis contribute to altered spinal biomechanics. Recognition of this process is crucial for surgical planning and for predicting natural stabilization in degenerative lumbar conditions. Image created using the Mind the Graph platform (www.mindthegraph.com), account paid by the corresponding author E.R.V. The original schematic has not been reproduced from previous publications, and no third-party permissions were required. No artificial intelligence tools were used in the generation or the writing of this article; the diagrams were completely conceptualized and designed by the authors. Abbreviations: DS, degenerative spondylolisthesis.

The facet joints have recently received considerable attention as significant contributors to lumbar stability and biomechanics (see Figure 4 for the pathophysiological mechanisms of facet joint fusion). Their orientation, hypertrophy, and degeneration influence the choice of surgical approach and the likelihood of solid arthrodesis. Facet joint fusion has emerged as a valuable adjunct to interbody and posterolateral techniques, providing an additional site for bony union and contributing to overall segmental rigidity [9,10,33].

Radiological assessment is vital in the pre- and postoperative periods. Preoperative radiological assessment includes assessment of vertebral slippage using plain X-rays and MRI, as well as CT to evaluate neural foraminal and facet joint assessment and degeneration. Flexion-extension radiographs (X-rays) are the standard for detecting dynamic spinal instability. At the same time, MRI is the predominant method for evaluating the spinal soft structures, neural elements, and the overall clinical condition of the neural elements, thereby determining the extent of surgical decompression of the spinal cord required [1,4].

It is essential to evaluate postoperative status to assess fusion progression and to optimize future decision-making (Table 3).

**Table 3:** Imaging modalities for assessing lumbar fusion - radiography, CT, MRI, PET/CT, and SPECT/CT. Comparative overview of the principal imaging modalities used to evaluate lumbar fusion, highlighting diagnostic advantages, limitations, sensitivity, specificity, and typical postoperative timelines for optimal assessment of spinal fusion integrity. Sensitivity and specificity ranges represent pooled estimates across the cited systematic reviews and primary diagnostic-accuracy studies. Source: Adapted by the authors from references [34-37]. Abbreviations: CT, computed tomography; MRI, magnetic resonance imaging; PET/CT, positron-emission tomography combined with computed tomography; SPECT/CT, single-photon emission computed tomography combined with computed tomography.

Imaging modality	Advantages	Limitations	Sensitivity/Specificity	Optimal postoperative timing
Radiography	Widely available; low cost; dynamic (flexion-extension) views for instability assessment	Limited for detecting early fusion; difficulty visualizing posterior elements and cage integration	~60-70% / ~65-75%	Early (6-12 weeks) for hardware alignment; 12 months for fusion signs
CT	High-resolution bone detail; excellent visualization of graft incorporation and hardware position	Higher radiation dose; may overestimate pseudarthrosis owing to metal artifact	~85-95% / ~90-95%	6-12 months for evaluating solid fusion
MRI	Superior soft-tissue contrast; evaluates neural structures and adjacent segment disease	Metal artifacts from instrumentation; suboptimal for bony fusion assessment	~75-85% / ~80-90%	After 6 months for soft-tissue and neural assessment
PET/CT	Functional assessment of metabolic activity; differentiates infection vs. nonunion	High cost; limited availability; false positives in early healing	~90-95% / ~85-90%	>6 months when assessing for nonunion or infection
SPECT/CT	Localises symptomatic facet joints and discs; prognostic for surgical pain outcomes	Radiation; limited availability; requires specialised interpretation	~88-92% / ~80-88%	>6 months for preoperative planning and postoperative pain assessment

Conventional radiographs, particularly lateral views, are commonly used to evaluate bridging trabecular bone and hardware integrity; however, their sensitivity for detecting pseudarthrosis is limited. Thin-slice CT with multiplanar reconstructions offers superior visualization of bony fusion within interbody and facet joint spaces and is considered the gold standard for radiographic assessment of fusion [7,8,37].

**Figure 5:**
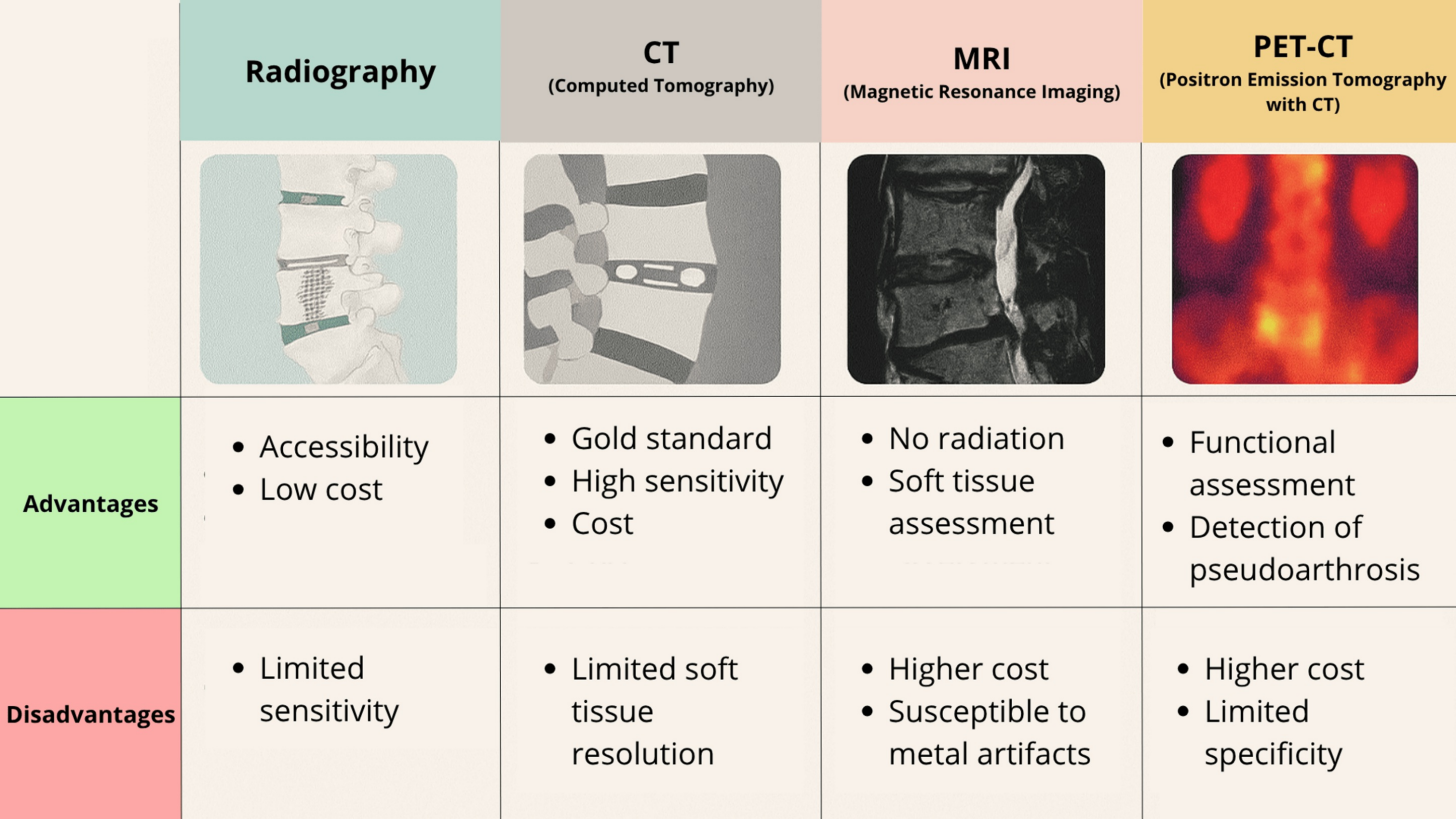
Imaging modalities for assessing postoperative lumbar fusion. Comparative overview of the advantages and limitations of radiography, CT, MRI, and PET/CT in the assessment of lumbar spinal-fusion status. CT remains the gold standard for detecting solid fusion; MRI helps in evaluating soft-tissue healing and adjacent segment disease; functional imaging techniques such as PET/CT show promise for identifying metabolic activity indicative of pseudarthrosis or ongoing fusion. Image created by the authors using the Mind the Graph platform (www.mindthegraph.com); original schematic not reproduced from previous publications; no third-party permissions required. Abbreviations: CT, computed tomography; MRI, magnetic resonance imaging; PET/CT, positron-emission tomography combined with computed tomography.

Advanced modalities such as positron emission tomography/computed tomography (PET/CT) and dynamic contrast-enhanced MRI have been explored to evaluate metabolic activity at the fusion site, particularly in equivocal cases where standard imaging is inconclusive. These tools may help distinguish delayed union, pseudarthrosis, and ongoing healing [5,35]. Single-photon emission CT/CT (SPECT-CT) has also gained traction as a functional adjunct to localize symptomatic facet joints and predict postoperative pain outcomes after lumbar interbody fusion, with disc-space uptake patterns associated with superior pain relief at 1 year [36]. See Figure 5 for imaging modalities in fusion assessment.

Understanding the anatomy of the facet joints and the morphology of the pelvis and spine is critical to surgical planning. Inadequate balancing of the spine and pelvis, and facet joint malalignments, lead to undesirable outcomes and degeneration of adjacent spinal joints. This knowledge emphasizes the need to focus on both the operative and surgical techniques selected for the procedure [3,22]. In summary, the integration of thorough radiological imaging assessment and anatomical knowledge has the potential to improve surgical practice, accurately identify anatomical structures, and elucidate surgical complications and outcomes. This approach is particularly the case for the facet joints, which, to a significant degree, have been the focus of fusion surgery and imaging.

WHICH: optimal surgical techniques and radiological methods

The optimal lumbar fusion surgical technique for DS requires a balance among the patient’s unique anatomy, clinical presentation, and the surgeon’s expertise. When considering interbody fusion techniques, no method in isolation (i.e., ALIF, OLIF, XLIF, PLIF, or TLIF) is inherently better than the others across all patients. The selection is dictated by the specific region’s biomechanical and pathological demands [2,7].

Surgical Techniques: Comparative Insights

Techniques should not be chosen based on the literature on fusion rates, as the results will often be the same when fusion occurs. Corridors, decompression mechanisms (direct vs. indirect), and postoperative profiles (i.e., blood loss, length of stay, and complications) account for the most variation among techniques.

Posterior lumbar interbody fusion and TLIF are widely favored for their ability to achieve 360-degree fusion by addressing the anterior and posterior columns. They permit disc-space restoration, correction of sagittal imbalance, and indirect decompression of neural elements. TLIF is valued for its unilateral approach, which minimizes dural retraction and the risk of neural injury [7]. ALIF, by contrast, provides unparalleled access to the anterior disc space and facilitates the placement of larger interbody cages, offering robust support for restoration of segmental lordosis. It carries its own risks, however, including vascular injury and retrograde ejaculation in male patients due to sympathetic plexus disruption [4]. OLIF provides preservation of the psoas muscle and avoids transection of the lumbar plexus but may cause abdominal or vascular injury and exhibits a higher overall complication rate when compared with ALIF (18.8% vs. 7.3%), driven mainly by cage subsidence rather than failed fusion [28].

Compared with TLIF, OLIF offers reduced blood loss, a shorter hospital stay, and better restoration of disc and foraminal height. TLIF is more commonly associated with dural tears, cerebrospinal fluid leakage, and nerve-root manipulation, whereas OLIF more often produces transient thigh symptoms, sympathetic-chain irritation, and hip-flexor weakness; OLIF is preferred when indirect decompression is the goal [18].

Oblique lateral interbody fusion can be used for a wider range of patients in terms of vascular architecture, lumbar plexus positioning, psoas morphology, iliac crest height, and the level of the spine to be treated [26].

Posterolateral fusion is particularly useful when facet joint degeneration is expansive or when interbody access is limited. It provides a less invasive means of achieving fusion through decorticating the transverse processes and placing grafts in the posterolateral gutters. Fusion rates with PLF alone, however, have been reported to be lower than those achieved with interbody techniques, particularly in patients with significant instability [8,9,22].

Role of Facet Joint Fusion

The incorporation of facet joint fusion (FJF) is increasingly of interest in the surgical management of degenerative segmental instability. Although fusion of the facet joints has historically been considered an incidental finding, it is now believed to improve segmental stabilization and, consequently, fusion rates. Initiatives that focus on facet joint surface decortications with grafting of the joint space promote bone union. Studies have shown that combined facet joint fusion has led to substantial clinical improvement and reduced adjacent segment disease, which is considered an important addition to the surgical treatment of DS [9-11,33].

Recent prospective data after lateral lumbar interbody fusion (LLIF) demonstrate that spontaneous facet fusion occurs in approximately 65% of facet joints and frequently precedes interbody fusion, with preoperative facet osteoarthritis and solid interbody fusion as significant predictors [38]. These findings reinforce the clinical relevance of monitoring facet joint fusion as a marker of segmental healing rather than treating it as an incidental finding. In comparative studies of OLIF (versus TLIF, PLIF, XLIF, and others), final fusion rates are typically in the 90-100% range, comparable to other interbody techniques, including anterior approaches [18,30]. Emerging high-quality evidence continues to refine the role of the facet joint in fusion surgery: a recent double-blinded, randomized, placebo-controlled trial showed that an intraoperative facet joint block reduces early postoperative pain and opioid consumption after OLIF [39], and a 2025 systematic review and meta-analysis found that adding fusion to decompression for facet-related pathology lowered recurrence and improved back-pain resolution [40]. Consistent with this nuance, an institutional CT-mapping study of 360° TLIF for DS documented high interbody and facet joint fusion rates, yet the anatomical distribution and number of fused zones did not correlate with 12-month Oswestry Disability Index or Roland-Morris scores, indicating that fusion location alone does not determine clinical outcome [41]. More recently, a 2026 cohort of posterior C1-2 fusion confirmed that spontaneous facet (auto)fusion is closely linked to preoperative facet osteoarthritis and contributes to overall radiographic stability - an observation that parallels the role of spontaneous facet fusion increasingly recognized in the lumbar spine [42].

Radiological Methods: Evaluating Fusion Success

Radiographic confirmation of fusion remains a cornerstone of postoperative assessment. Plain radiographs are widely accessible but offer limited sensitivity for detecting pseudarthrosis and are prone to observer variability. Dynamic flexion-extension films can demonstrate segmental motion, but their ability to confirm solid arthrodesis is debated [1,34].

Computer tomography imaging with thin-slice acquisition and multiplanar reconstruction has emerged as the gold standard for evaluating interbody and posterolateral fusion. It enables detailed visualization of bony trabeculation across fusion sites and identifies hardware-related complications such as screw loosening or breakage. In cases of uncertainty regarding pseudarthrosis, advanced techniques such as single-photon emission computed tomography (SPECT)/CT and PET/CT may provide additional insight by detecting metabolic activity indicative of nonunion or ongoing fusion [5,35-37].

Recent investigations into radiomics and artificial intelligence (AI)-driven imaging analysis show promise for improving fusion assessment accuracy. Deep-learning radiomics models that integrate preoperative CT, multi-sequence MRI, and clinical features have outperformed expert surgeons for predicting cage subsidence after lumbar fusion, suggesting earlier detection of fusion failure and improved long-term risk stratification [43]. Conventional CT-based studies similarly identify cage geometry, vertebral Hounsfield units, and BMP use as independent predictors of subsidence and pseudarthrosis [44].

Clinical Decision-Making Considerations

Proper selection of both surgical and imaging modalities is patient-specific. The decision must incorporate many factors, including the patient’s age, bone quality (e.g., osteoporosis), severity of spondylolisthesis, spinopelvic alignment, and other medical conditions. For example, elderly patients with poor bone stock may benefit from augmented fixation strategies, such as cement-augmented pedicle screws or bone morphogenetic protein (BMP) use, to enhance fusion rates [11,23,24]. Intraoperative O-arm navigation during MIS-TLIF reduces facet joint violation (23.8% vs. 53.6%) and delays supradjacent facet degeneration, thereby supporting the use of navigational technologies as the norm in modern-day practice [45]. Integrating surgical-technique selection with robust postoperative imaging protocols allows clinicians to confirm successful arthrodesis and intervene early if complications arise. Optimizing these decisions is crucial to achieving superior functional outcomes and minimizing the need for revision surgery.

HOW: practical clinical implementation, postoperative care, and long-term management

Management of DS involves careful planning that begins well before the surgical procedure and continues long after. While the surgical step may be the most prominent and easily recognizable element of a management plan, the thoughtful planning and implementation of measures to address the preoperative, postoperative, and long-term aspects of the management plan are equally important, if not more important, in maximizing the success of the surgical intervention.

Preoperative Considerations and Planning

The patient-first mentality starts with a thorough preoperative evaluation, allowing for improved stratification of surgical risk and the identification of factors that can be improved (Table 4).

**Table 4:** Postoperative management protocols to enhance lumbar fusion rates. Summary of the principal postoperative strategies aimed at optimizing spinal fusion outcomes, including immobilization techniques, rehabilitation protocols, pharmacologic adjuncts, and modification of identified risk factors. The level of supporting evidence varies between strategies, with the strongest data supporting smoking cessation, glycemic control, and selected biologic adjuncts. Source: Adapted by the authors from references [15,23,24,29,31,46]. Abbreviations: BMI, body mass index; BMP, bone morphogenetic protein; TLSO, thoraco-lumbo-sacral orthosis; D3, vitamin D3 (cholecalciferol); K2, vitamin K2 (menaquinone).

Strategy	Description	Evidence / Considerations
Immobilization	Use of rigid or semi-rigid lumbar orthoses (e.g., TLSO braces) during the early healing phase	Supports fusion-site stability; controversial efficacy; typically used for 6-12 weeks postoperatively
Rehabilitation protocols	Gradual mobilization, core strengthening, and physiotherapy initiated within 4-6 weeks postoperatively	Early controlled activity promotes recovery; excessive early motion may compromise fusion
Bone morphogenetic proteins (BMPs)	Osteoinductive agents applied during surgery to stimulate bone formation	Proven to enhance fusion rates, particularly in high-risk patients; potential for increased inflammation and cost
Bisphosphonates	Anti-resorptive agents to prevent bone loss in osteoporotic patients	Mixed evidence on efficacy; may inhibit remodeling if used too early after surgery
Vitamin K2 + D3 supplementation	Combined K2 (45 mg/day) and D3 (250 IU/day) with calcium in osteoporotic patients	Recent RCT shows higher fusion rates at 6 months (91.7% vs. 74.3%) in endoscopic lumbar interbody fusion
Smoking cessation	Elimination of tobacco use to improve bone healing	Strongly associated with improved fusion rates; nicotine is a known inhibitor of osteogenesis
Nutritional optimization	Adequate protein, calcium, and vitamin D supplementation	Critical for bone metabolism; deficiencies are linked to higher pseudarthrosis risk
Diabetes control	Strict glycemic management in diabetic patients	Hyperglycemia impairs osteoblastic activity and increases infection risk

Particularly for geriatric and frail patients, vital sign prehabilitation, such as primary prevention focused on improving the strength and control of the core and increasing cardiovascular endurance, as well as patient education, may be helpful if implemented before surgery and may enhance the recovery process. Planning the surgery is guided by imaging, especially diagnostic radiology performed with the patient standing, in conjunction with MRI, which can show compression of the neural elements and the status of the facet joints, as well as sagittal-plane spinal balance, which may be lost [1,3,15].

In patients with poor bone quality, augmentation techniques such as BMPs, hydroxyapatite, or cement-augmented screws can enhance fusion potential and construct stability [23,24,46]. Shared decision-making with the patient regarding the extent of surgery (decompression alone vs. decompression with fusion) is also crucial, especially in borderline cases where instability is subtle [4].

Postoperative Care and Early Rehabilitation

During the immediate postoperative period, the focus is largely on pain control, neurologic monitoring, and prevention of complications such as surgical site infection, deep vein thrombosis, and pulmonary compromise. Multimodal analgesia, including regional techniques such as the erector spinae plane block, can reduce opioid consumption and facilitate early mobilization.

Bracing remains a debated adjunct. Many believe that lumbar orthoses should be used to limit segmental movement and fuse the segments. In contrast, others largely refute these claims due to a lack of evidence supporting their effectiveness. However, careful mobilization protocols tailored to the surgical approach (e.g., ALIF vs. TLIF) are essential to promote functional recovery without jeopardizing the fusion construct [7,31].

To heal the spine, the focus should be on optimizing the patient’s nutrition, with adequate calcium and vitamin D supplementation to support bone healing. In osteoporotic patients, combined vitamin K2 and D3 supplementation has been shown to improve early fusion rates after endoscopic lumbar interbody fusion (91.7% vs. 74.3% at six months), suggesting that micronutrient optimization is more than supportive care [29]. Early engagement of physical therapy, focused on gentle range-of-motion exercises and gradual strengthening, can expedite recovery while minimizing adjacent-segment stress.

Radiological and Clinical Follow-Up

Postoperative imaging is one of the most important tools for providing the surgeon with information on the current state of the fusion and the presence of any complications. The standard of care in this procedure includes plain imaging at defined intervals (6 weeks, 3 months, 6 months, and 1 year), with CT reserved for nonunion or for imaging that shows the patient still has symptoms of the surgery. Advanced modalities, including PET/CT and SPECT/CT, may be used in complex cases to differentiate pseudarthrosis from infection or mechanical pain [8,35-37].

From a clinical standpoint, patient-reported outcome measures (PROMs), such as the Oswestry Disability Index (ODI) and the visual analog scale (VAS) for pain, should be tracked longitudinally to assess functional recovery and quality of life. Close monitoring for adjacent-segment disease, a known long-term complication of fusion, enables timely intervention.

Long-Term Management and Secondary Prevention

The long-term strategy needs to prioritize maintaining spinal health and preventing the recurrence and/or progression of pathology. The use of strengthening programs for paraspinal and core muscles should reduce the stress on adjacent spinal segments. Bone-health monitoring in older adults is critical, with pharmacologic intervention (e.g., bisphosphonates or denosumab) considered for osteoporosis to prevent vertebral fractures that could destabilize the construct [12,46]. A 2026 systematic review and meta-analysis further indicated that fusion approaches that preserve the paraspinal musculature may be associated with lower rates of adjacent-segment disease and related reoperations. However, prospective confirmation is still required [47].

There are several areas of fusion surgery that are, as of now, the realm of speculation and/or research. Traditional radiology will be augmented by AI-based algorithms capable of predictive analytics to assess the success of spinal fusion and the risk of complications. Integration of these tools into clinical workflows may enable truly personalized postoperative management strategies in the near future [43].

Multidisciplinary Coordination

Optimal postoperative care involves coordinated input from spine surgeons, radiologists, pain specialists, physiotherapists, and primary care physicians. This multidisciplinary collaboration ensures comprehensive care that addresses the biomechanical, neurologic, and psychosocial dimensions of recovery. The “HOW” of lumbar fusion in DS therefore encompasses a continuum that begins with careful patient selection and extends through structured postoperative protocols and vigilant long-term follow-up.

Key clinical insights

The pathophysiology of degenerative spondylolisthesis (DS) involves disc degeneration, facet joint, and ligament degeneration.Understanding these mechanisms may enable better identification of patients for fusion and the selection of more suitable approaches. Notably, facet joint fusion of the lumbar spine is a significant yet commonly unrecognized factor in the successful treatment of DS and other spinal pathologies.

Impact of Facet Joint Fusion on Postoperative Outcomes: Long-term studies of instrumented facet fusion and spontaneous fusion of facet joints in patients following LLIF support the assertion that facet joint fusion is integral to the stabilization of the spine and is likely to result in improved postoperative outcomes, especially in the older population with compromised bone quality [9,10,33,38]. Framed through the Who, Why, What, When, Where, Which, and How structure, this review identifies facet joint fusion as a key, often underrecognized, determinant of treatment success and maps the principal factors for optimizing patient care (Table 5).

**Table 5:** Summary of the WHO-WHY-WHAT-WHEN-WHERE-WHICH-HOW framework mapped to key clinical recommendations. This summary table links each domain of the structured framework used in this review to a concise, evidence-based clinical recommendation for lumbar facet joint fusion in degenerative spondylolisthesis (DS). Abbreviations: ALIF, anterior lumbar interbody fusion; OLIF, oblique lateral interbody fusion; PLF, posterolateral fusion; PLIF, posterior lumbar interbody fusion; TLIF, transforaminal lumbar interbody fusion; XLIF, extreme lateral interbody fusion.

Domain	Focus	Key clinical recommendation
WHO	Patient characteristics and clinical presentation	Identify candidates most likely to benefit (typically older adults with DS and mechanical or claudicant symptoms) and optimize modifiable comorbidities - osteoporosis, diabetes, and smoking - before surgery.
WHY	Pathophysiology and biomechanics	Aim to restore segmental stability and relieve neural compression, with particular attention to the L4-L5 segment where DS predominates.
WHAT	Surgical techniques and fusion strategies	Select the interbody or posterolateral technique (ALIF, OLIF, XLIF, PLIF, TLIF, or PLF) according to patient anatomy and goals, and interpret reported fusion rates in light of methodological heterogeneity.
WHEN	Indications and timing	Base the decision on the whole clinical picture - persistent symptoms, demonstrable instability, and failure of conservative care - rather than on a single radiographic threshold.
WHERE	Anatomy and radiological assessment	Use MRI and CT for preoperative planning and thin-slice CT for postoperative fusion assessment, with explicit evaluation of the facet joints.
WHICH	Optimal surgical and imaging methods	Match the surgical technique and imaging modality to the individual patient; CT remains the reference standard for confirming fusion, with radiomics and AI tools emerging.
HOW	Implementation and long-term management	Apply multimodal pain control, early tailored rehabilitation, risk-factor modification, and multidisciplinary follow-up to maximize fusion and limit adjacent-segment disease.

Surgical Decision-Making and Fusion Rates: The choice between decompression alone and decompression with fusion should be guided by patient-specific factors, including dynamic instability, degree of slippage, and comorbidities. Current network meta-analyses indicate broadly similar pain and disability outcomes across decompression-only, dynamic-stabilization, and fusion strategies, but with meaningful differences in operative time and blood loss [5,6,8,32].

Role of Imaging in Preoperative and Postoperative Phases: Cross-sectional modalities (MRI, CT) are essential for surgical planning and for assessing fusion progression; PET/CT and SPECT/CT are valuable adjuncts when standard radiographs are inconclusive, with SPECT-CT uptake patterns offering prognostic value for surgical outcomes [8,35-37].

Optimizing Postoperative Recovery: Early mobilization, multimodal pain control, and tailored rehabilitation are pivotal; nutritional optimization (including combined vitamin K2 and D3 in osteoporotic patients) and pharmacologic support for bone health are equally important in promoting successful fusion [29,31,46].

Long-Term Monitoring and Secondary Prevention: There is a need for follow-up to detect adjacent segment disease, hardware complications, and delayed fusion. Incorporating PROMs and periodic imaging into long-term care ensures a proactive approach to preserving spinal function and quality of life.

Future Directions and Innovation: Radiomics, artificial intelligence, biportal endoscopic approaches, and robotic ALIF hold promise for personalized, less invasive, and more accurately predicted lumbar spine care over the coming decade [16,21,43].

## Conclusions

Having a clear understanding of patient characteristics and clinical presentations (WHO) permits the surgeon to integrate more thoughtful planning prior to surgery and helps to locate the patients that are most likely to reap the benefits of fusion. Moreover, having some awareness of the biomechanical factors and some of the pathophysiologic processes responsible for degenerative spondylolisthesis (WHY) is equally important. In this case, the surgeon will appreciate the need for restoration of stability and the alleviation of neural compression. In addition, getting a full understanding of the surgical practices and fusion techniques (WHAT) with some logic as to the appropriate time and indications (WHEN) will assist the surgeon in reducing postoperative complications. The complexities of the anatomy of facet joint involvement and the use of imaging to evaluate fusion (WHERE) highlight the importance of careful and exhaustive evaluation prior to surgery and postoperatively. Choosing the best surgical techniques and imaging modalities (WHICH), along with the development of strategies to enhance outcomes (HOW), will continue to be a primary concern for the surgeon.

Achieving solid facet joint and interbody fusion appears to be associated with improved functional recovery, reduced pain, and lower reoperation rates; however, much of the available evidence regarding facet joint fusion remains observational, and its independent contribution to long-term outcomes should be confirmed in prospective studies. For clinicians and surgeons, maintaining a high index of suspicion for fusion failure, leveraging multimodal imaging tools, and adopting multidisciplinary care pathways are pivotal to enhancing treatment success. As spinal-surgery techniques and technologies evolve, future research should continue to refine fusion strategies and clarify their impact on quality of life across diverse patient populations. This review presents a clinically relevant synthesis for physicians and surgeons who aim to improve the precision and efficacy of lumbar fusion interventions in daily practice.
